# The Changes of Irisin and Inflammatory Cytokines in the Age-Related Macular Degeneration and Retinal Vein Occlusion

**DOI:** 10.3389/fendo.2022.861757

**Published:** 2022-03-17

**Authors:** Xiaochun Li, Xiaoguang Cao, Mingwei Zhao, Yongzhen Bao

**Affiliations:** ^1^ Department of Ophthalmology, Peking University People’s Hospital; Eye Diseases and Optometry Institute; Beijing Key Laboratory of Diagnosis and Therapy of Retinal and Choroid Diseases; College of Optometry, Peking University Health Science Center, Beijing, China; ^2^ Department of Ophthalmology, Peking University International Hospital, Beijing, China

**Keywords:** irisin, age-related macular degeneration (AMD), retinal vein occlusion (RVO), macular thickness, inflammatory factor

## Abstract

**Purpose:**

Age-related macular degeneration (AMD) and retinal vein occlusion (RVO) are irreversible chorioretinal diseases, which might induce severe damage in visual function. The metabolic factor and inflammatory factors might play important roles in the pathogenesis of AMD and RVO. The levels of irisin and 14 cytokines were analyzed in aqueous humor of AMD and RVO eyes to evaluate the roles of irisin and inflammatory factors.

**Methods:**

We collected aqueous humor samples from patients with AMD (*n* = 27), RVO (*n* = 30), and cataract (as control, *n* = 23) eyes. Samples were assayed using ELISA kit for irisin and a multiplex immunoassay kit for 14 cytokines. The macular thickness (MT) was measured with OCT in all included eyes.

**Results:**

MT in the RVO group is significantly higher than that in the AMD or control group. Irisin levels in the aqueous samples of AMD and RVO eyes were both significantly lower than that in the control. Furthermore, a positive correlation was found between irisin and MT in the RVO. Compared with the controls, AMD eyes had significantly higher levels of BDNF, VEGF-A, VEGF-R1, VEGF-R2, IL-10, TNF-α, VCAM-1, IP-10, and MCP-1. Similarly, RVO eyes had significantly higher levels of BDNF, VEGF-A, VEGF-R1, VEGF-R2, IL-6, IL-8, IL-10, TNF-α, ICAM-1, VCAM-1, IP-10, and MCP-1. However, there was no significant difference between the levels of PDGF-BB or TNF-β in these three groups. A negative correlation was found between VEGF-A and MT in AMD, as well as in control. Furthermore, a positive correlation was found between IL-6 and MT in the 80 included eyes, as well as in RVO. A positive correlation was found between ICAM-1 and MT in the 80 included eyes, as well as in RVO.

**Conclusions:**

The metabolic factor, irisin levels in the aqueous humor are decreased in AMD and RVO eyes and show a positive correlation between irisin and MT in RVO eyes, prompting researchers to explore the relationship between irisin and macular edema. We also identified the higher expression of vascular growth factors (VEGF-A, VEGF-R1, and PDGF-BB), inflammatory cytokines (IL-6, IL-8, IL-10, and TNF-α), and chemokines (ICAM-1, VCAM-1, IP-10, and MCP-1) in AMD and RVO eyes.

## Introduction

In human sensory organs, the eye is the primary means to obtain information. With the development of technology, many parts of human eyes, such as the cornea and lens, have artificial substitutes, but there is no substitute for the retina. The retina is a nerve tissue that is responsible for converting light signals into nerve signals, which are then transmitted to the visual center of the brain by the optic nerve (1). Various retinal diseases may seriously damage visual function and even lead to blindness (2).

With the process of aging society, age-related macular degeneration (AMD) has become one of the leading causes for inevitable blindness in China and worldwide, as an estimated 288 million people worldwide will suffer from AMD by 2040 (3, 4). The etiology of AMD is complex and several possible risk factors for the development of AMD had been reported, the major one being aging, and others such as oxidative stress and inflammatory damage. Moreover, the association between AMD and some systemic disorders has also been reported. Previous studies have shown a significant link between AMD occurrence and diabetes, obesity, high body mass index, hypertension, coronary heart disease, dyslipidemia, chronic kidney disease, and Alzheimer’s disease (5–7).

Retinal vein occlusion (RVO) is caused by retinal vein thrombosis and affects 16 million people worldwide. It is the second most common retinal disease after diabetic retinopathy and may be related to severe consequences such as neurovascular glaucoma, retinal detachment, and eventual blindness (8). As categorized according to the site of vascular occlusion, RVO includes branch RVO (BRVO), central RVO (CRVO), and hemi-RVO (9). As the occurrence of hemorrhages and macular edema (ME), RVO could lend to significant visual impairment (10). Several common cardiovascular risk factors, such as hypertension, diabetes, and hyperlipemia, were reported to be predisposing factors for RVO and to enhance the risk of RVO recurrence (11, 12).

Irisin is a cleavage product of fibronectin type III domain-containing protein 5 (FNDC5) and can act on white adipocytes and induce them to transform into brown adipocytes. Irisin exists in various organs, including brain, heart, liver, and skeletal muscle and was reported to control glucose metabolism, lipid metabolism and energy homeostasis in skeletal muscle and adipose tissue (13–16). In addition, a recent study showed that irisin had anti-inflammatory, antioxidant, and anti-apoptotic effects (17). Irisin could cross the blood–brain barrier, and its expression had been found in some sites of the central nervous system (CNS) (18, 19). Previous studies had shown that irisin could enhance the expression of brain-derived neurotrophic factor (BDNF) in various brain regions with subsequent beneficial effects on brain health and cognitive function (20, 21).

One previous study reported that irisin could be detected in the retinae and other studies reported the level of irisin in the aqueous humor of cataract with/without pseudoexfoliation, and high myopia (22–25). Moreover, the concentration of serum irisin was lower in those patients with type 2 diabetes mellitus, and was associated with the presence of diabetic nephropathy and diabetic retinopathy (26). The goals of our study are to identify the expression of irisin in AMD and RVO and to explore its role and potential application in those ocular diseases. On the other hand, previous studies have reported the association of AMD and RVO with inflammatory stress; the relationship of irisin and inflammatory cytokines is also explored.

## Methods

### Study Design

Twenty-seven eyes from 27 patients with AMD, thirty eyes from 30 patients with RVO, and twenty-three eyes from 23 patients with cataract were studied from December 2020 to September 2021, and comprise the AMD group, RVO group, and control group, respectively. All included AMD and RVO eyes were planned to have an uneventful intra-vitreal anti-VEGF therapy. The inclusion criterion for those patients with AMD was diagnosed with AMD, exclusive of any other retinal disorder. The inclusion criterion for those patients with RVO was diagnosed with RVO, exclusive of any other retinal disorder. The inclusion criterion for those patients with cataract was an uneventful cataract surgery. Eyes with glaucoma, uveitis, zonular weakness, previous trauma, previous intraocular surgery, or fundus pathology were excluded from the study. Patients with diabetes mellitus were excluded. The study was approved by the Human Research Ethics Committee of Peking University People’s Hospital and Peking University International Hospital, and adhered to the guidelines of the Declaration of Helsinki. Written informed consent was obtained from all included patients.

### Sample Collection

We administered Oxybuprocaine Hydrochloride Eye Drop (Santen Pharmaceutical Co., Ltd., Osaka, Japan) to the patients 4 times every 5 min before the surgery as the local anesthesia. Eyelids and the surrounding skin were swabbed with povidone iodine. Samples of aqueous humor (90–120 μl) were aspirated by inserting a 29-gauge needle through the corneal paracentesis into the anterior chamber before surgery. Samples were immediately stored at −80°C until sample analysis.

### Irisin Analysis

Samples were harvested and assayed using the enzyme-linked immunosorbent assay (ELISA) kit for irisin (Irisin ELISA Kit; Beijing Dongge Boye Biotechnology Co. Ltd., Beijing, China) and were measured according to the manufacturer’s instructions. The stop solution changes the color from blue to yellow and the intensity of the color is measured at 450 nm using a spectrophotometer. In order to measure the concentration of irisin in the sample, the Irisin ELISA Kit includes a set of calibration standards. The calibration standards are assayed at the same time as the samples and allow the operator to produce a standard curve of optical density (OD) compared to irisin concentration. The concentration of irisin in the samples is then determined by comparing the OD of the samples to the standard curve.

### Cytokine Analysis

We simultaneously analyzed a selection of 14 cytokines, namely BDNF, intercellular cell adhesion molecule-1 (ICAM-1), interleukin (IL)-10, IL-8, IL-6, inducible protein-10 (IP-10), monocyte chemoattractant protein-1 (MCP-1), platelet-derived growth factor BB (PDGF-BB), tumor necrosis factor α (TNF-α) and β (TNF-β), vascular cell adhesion molecule-1 (VCAM-1), vascular endothelial growth factor (VEGF), and vascular endothelial growth factor receptor 1 (VEGF-R1) and 2 (VEGF-R2). Cytokine concentrations were quantified in duplicate using a magnetic bead 14-plex panel assay (Human ProcartaPlex Mix&Match 14-plex, PPX-14-MX9HJ62; Thermo Fisher Scientific, Waltham, USA) performed according to the manufacturer’s recommended protocol and read using a Bio-Plex 200 array reader (Bio-Rad, Hercules, California, USA). The standard curve was based on five-parameter nonlinear regression. Each cytokine concentration was then calculated by the curve.

### Measurement of Macular Thickness

All included eyes were measured using a spectral-domain OCT (3D OCT-1, Topcon, Tokyo, Japan) without dilating the pupil. The macula full retinal thickness was defined as the distance between the inner limiting membrane and the outer segments/retinal pigment epithelium junction boundary. It was divided into nine quadrants based on the ETDRS (Early Treatment Diabetic Retinopathy Study) map. The value of average thickness read from the OCT report was set as the macular thickness (MT).

### Statistical Analysis

The data were processed and statistically analyzed using IBM SPSS Statistics for Windows, v. 20.0 (IBM Corp., Armonk, USA). All data are presented as means ± standard deviations (SD). Categorical data were compared between groups using *χ^2^* test. Student’s *t*-test and Mann–Whitney *U* test were used to detect differences between AMD, RVO, and control groups. Pearson’s correlation analysis was adopted to analyze the relationships among the cytokines and MT in our study. Values of *p* < 0.05 were considered statistically significant.

## Results

### Baseline Characteristics of the Participants

The distributions of age, gender, and eye are summarized in **Table 1**. All included AMD eyes were wet-AMD. The included 30 RVO eyes had 13 eyes with BRVO and 17 eyes with CRVO. There was a significant difference in age and no significant difference in gender or eye in the three groups.

**Table 1:** Baseline characteristics of the patients in respective groups.

Group (*n* = 80)	Age (years)	Gender	Eye	Macular thickness (MT) (um)
AMD (*n* = 27)	71.07 ± 8.77	Male (*n* = 19) 70.37% Female (*n* = 8) 29.63%	Right eye (*n* = 12) 44.44% Left eye (*n* = 15) 55.56%	275.20 ± 27.83
RVO (*n* = 30)	59.40 ± 14.52$	Male (*n* = 18) 60.00% Female (*n* = 12) 40.00%	Right eye (*n* = 12) 40.00% Left eye (*n* = 18) 60.00%	359.61 ± 87.39$
Control (*n* = 23)	71.78 ± 13.27	Male (*n* = 13) 56.52% Female (*n* = 10) 43.48%	Right eye (*n* = 15) 65.22% Left eye (*n* = 8) 34.78%	265.66 ± 16.89
*p*-value	0.000	0.07	0.293	0.000

The difference in age and macular thickness (MT) between the three groups was tested using one-way ANOVA. The difference in age and MT between AMD and control groups, and that between the RVO and control group were tested using independent Student’s t-test, and $ p < 0.05. The difference in gender and eye distribution between the three groups was tested using χ^2^ test.

### Macular Thickness Measurement

The measurement of MT is shown in **Table 1**. MT in the RVO groups is higher than that in the AMD group (*t* = −5.016, *p* = 0.000) or the control group, significantly (*t* = 5.75, *p* = 0.000). There is no significant difference in MTs between the AMD and control groups.

### Irisin Levels in the Aqueous Humor of the Study Eyes

The irisin levels in the aqueous samples of AMD, RVO, and control groups are shown in **Table 2**. Student’s *t*-test revealed that irisin level in the aqueous samples of the AMD group was significantly lower than that of the control group (*t* = −3.357, *p* = 0.002). The irisin level of RVO group was similarly lower than that of the control group (*t* = −3.221, *p* = 0.002). Those of the AMD and RVO groups have no significant difference (*t* = 0.243, *p* = 0.809). Furthermore, a positive correlation was found between irisin and MT in the RVO group (Pearson = 0.468, *p* = 0.009).

**Table 2:** The levels of irisin, BDNF, VEGF-A, VEGF-R1, VEGF-R2, and PDGF-BB in the aqueous humor.

Group (*n* = 80)	Irisin	BDNF	VEGF-A	VEGF-R1	VEGF-R2	PDGF-BB
AMD (*n* = 27)	64.81 ± 24.40	1.26 ± 0.72	7,094.78 ± 5,961.80	4,650.89 ± 6,590.30	863.68 ± 428.65	6.10 ± 22.21
RVO (*n* = 30)	63.00 ± 30.92	0.97 ± 0.39	11,291.77 ± 14,146.21	1,732.77 ± 3,109.11^#^	1,023.11 ± 511.22	3.14 ± 12.13
Control (*n* = 23)	88.92 ± 26.34*^$^	0.67 ± 0.19*^$^	2,518.33 ± 2,098.66*^$^	435.28 ± 336.12*^$^	633.96 ± 327.34*^$^	1.24 ± 4.70

Brain-derived neurotrophic factor (BDNF), Vascular endothelial growth factor (VEGF), Vascular endothelial growth factor receptor 1 (VEGF-R1) and 2 (VEGF-R2), Platelet derived growth factor BB (PDGF-BB). Data are expressed as the means ± standard deviations (SD), in pg/ml. The differences in cytokines between the 2 groups were tested using Student’s t-test. *p < 0.05 between the AMD and control group; ^$^p < 0.05 between the RVO and control group; ^#^p < 0.05 between the AMD and RVO group.

### BDNF Levels in the Aqueous Humor of the Study Eyes

As shown in **Table 2**, the levels of BDNF of the AMD group and RVO group were higher than that of the control group significantly (*p* = 0.000 and *p* = 0.001, respectively). However, there is no significant difference between the levels of BDNF in AMD and RVO groups (*p* = 0.068).

### VEGF-A, VEGF-R1, VEGF-R2, and PDGF-BB Levels in the Aqueous Humor of the Study Eyes

As shown in **Table 2**, the levels of VEGF-A, VEGF-R1, and VEGF-R2 of the AMD group and RVO group were higher than that of the control group significantly (VEGF-A, *p* = 0.001 and *p* = 0.002; VEGF-R1, *p* = 0.003 and *p* = 0.031; VEGF-R2, *p* = 0.041 and *p* = 0.002, respectively). The level of VEGF-R1 in the AMD group was higher than that in the RVO group significantly (*p* = 0.034). However, there was no significant difference between the levels of PDGF-BB in these three groups. Furthermore, a positive correlation was found between VEGF-R2 and MT in the total 80 included eyes (Pearson = 0.339, *p* = 0.002). A negative correlation was found between VEGF-A and MT in the AMD group (Pearson = −0.435, *p* = 0.023) and also in the control group (Pearson = −0.479, *p* = 0.021).

### Interleukin 6, 8, and 10 levels in the Aqueous Humor of the Study Eyes

As shown in **Table 3**, the level of IL-10 in the AMD group was higher than that of the control group significantly (*p* = 0.013). The levels of IL-6, IL-8, and IL-10 in the RVO group were higher than those of the control group (*p* = 0.036, 0.007, and 0.003, respectively). Moreover, the level of IL-8 in the AMD group was lower than that in the RVO group (*p* = 0.029). Furthermore, a positive correlation was found between IL-6 and MT in the total 80 included eyes (Pearson = 0.429, *p* = 0.000), and also in the RVO group (Pearson = 0.508, *p* = 0.004).

**Table 3:** The levels of, IL-6, IL-8, IL-10, TNF-α, and TNF-β in the aqueous.

Group (*n* = 80)	IL-6	IL-8	IL-10	TNF-α	TNF-β
AMD (*n* = 27)	43.98 ± 73.26	13.33 ± 22.44^#^	1.37 ± 0.97	13.01 ± 10.84	0.82 ± 3.78
RVO (*n* = 30)	67.03 ± 120.68	45.54 ± 73.83	1.16 ± 0.46	10.85 ± 5.33	17.55 ± 96.14
Control (*n* = 23)	17.47 ± 23.54^$^	6.08 ± 5.93^$^	0.87 ± 0.17*^$^	6.53 ± 2.28*^$^	0.00 ± 0.00

Interleukin (IL), Tumor necrosis factor α (TNF-α) and β (TNF-β). Data are expressed as the means ± standard deviations (SD), in pg/ml. The differences in cytokines between the 2 groups were tested using Student’s t-test. *p < 0.05 between the AMD and control group; ^$^p < 0.05 between the RVO and control group; ^#^p < 0.05 between the AMD and RVO group.

### Tumor Necrosis Factor α and β levels in the Aqueous Humor of the Study Eyes

As shown in **Table 3**, the levels of TNF-α in AMD and RVO groups were higher than those of the control group (*p* = 0.005 and *p* = 0.000, respectively). However, there was no significant difference between the levels of TNF-β in these three groups.

### ICAM-1, VCAM-1, IP-10, and MCP-1 Levels in the Aqueous Humor of the Study Eyes

As shown in **Table 4**, the AMD group had significantly higher levels of VCAM-1, IP-10, and MCP-1, compared with the control group (*p* = 0.036, 0.045, and 0.010, respectively). Moreover, the RVO group had significantly higher levels of ICAM-1, VCAM-1, IP-10, and MCP-1, compared with the control group (*p* = 0.000, 0.002, 0.000, and 0.005, respectively). However, the AMD group had a lower level of ICAM-1 than that of the RVO group (*p* = 0.000). Furthermore, a positive correlation was found between ICAM-1 and MT in the total 80 included eyes (Pearson = 0.530, *p* = 0.000) and also in the RVO group (Pearson = 0.407, *p* = 0.026).

**Table 4:** The levels of ICAM-1, VCAM-1, IP-10, and MCP-1 in the aqueous.

Group (*n* = 80)	ICAM-1	VCAM-1	IP-10	MCP-1
AMD (*n* = 27)	1,678.00 ± 1,205.49^#^	857.90 ± 724.14	54.99 ± 101.81	1,368.95 ± 678.02
RVO (*n* = 30)	3,323.35 ± 1,824.91	849.62 ± 415.38	38.67 ± 25.37	1,335.95 ± 582.13
Control (*n* = 23)	1,162.56 ± 773.04^$^	525.30 ± 299.90*^$^	13.62 ± 8.77*^$^	977.97 ± 290.40*^$^

Intercellular cell adhesion molecule-1 (ICAM-1), Inducible protein-10 (IP-10), Monocyte chemoattractant protein-1 (MCP-1), Vascular cell adhesion molecule-1 (VCAM-1). Data are expressed as the means ± standard deviations (SD), in pg/ml. The differences in cytokines between the 2 groups were tested using Student’s t-test. *p < 0.05 between the AMD and control group; ^$^p < 0.05 between the RVO and control group; ^#^p < 0.05 between the AMD and RVO group.

## Discussion

As one of the leading cause of inevitable blindness, AMD is very important in the ocular clinic and research. Besides it, another retinal disease, RVO, could lead to severe ocular disorders and the loss of visual function. The cytokine signal pathway and possible treatment factors are interesting.

Almost 10 years ago, irisin was identified as an exercise-induced hormone and found to be synthesized in some different tissues of several species (27, 28). Its cellular form, called FNDC5, is proteolytic cleavaged to be the secret form, irisin, and released into the circulation, which could be found not only in skeletal muscle cells, but also in nerve tissues, such in some sites of human brain, Purkinje cells, paraventricular nucleus, and cerebrospinal fluid (29–31). Several previous studies had investigated the immunoreactivity of irisin in one type of rodent, dwarf hamsters (*Phodopus roborovskii*), and irisin was found almost in all layers of retinae, except the outer nuclear layer (23). Moreover, the immunoreactivity of irisin was observed in the cornea (23). Another study of crested porcupine (*Hystrix cristata*) showed the immunoreactivity of irisin in the neural retina (24). Recently, two studies identified that irisin could be detected in the human aqueous humor and one study found that it could be detected in the vitreous humor (22, 25, 26). In our study, the AMD and RVO groups have significantly lower irisin in the aqueous than the control group. It could be that irisin is a retinal injury marker. As a positive correlation was found between irisin and MT in the RVO group, irisin could quantitatively reflect the MT in retinal disorders, especially in retinal vascular diseases.

In the pathogenic process of AMD, VEGF plays a very important role. Bis-retinoid N-retinyl-N-retinylidene ethanolamine (A2E) is found to accumulate in the retinae as aging and may induce oxidative damage (32) and cell damage and then finally stimulate cell death (33). In RPE cells, A2E was found to increase the level of VEGF mRNA and protein but did not affect the expression of VEGF-R1 or VEGF-R2. However, blue light exposure in A2E-loaded cells stimulated VEGF-R1 but not the levels of VEGF mRNA and protein (34). Currently, the intra-vitreal injection of anti-VEGF drugs has already become the standard treatment for wet-AMD (35). VEGF has been implicated in the etiology of various diseases of the retina including diabetic retinopathy (DR) and RVO. The use of anti-VEGF intra-vitreal injections has also profoundly impacted the treatment and visual outcomes in many of these entities, including macular edema (ME) secondary to RVO (36). Our study showed the level of VEGF-A in the aqueous of AMD and RVO groups to be higher than that in the control group, which also identified the role of VEGF in the pathogenesis of AMD and RVO, and the necessity of anti-VEGF treatment. Besides VEGF-A, the higher levels of VEGF-R1 in the AMD and RVO groups report of previous and identified VEGF-R1 could be a candidate molecular target whose suppression could supplement VEGF neutralization for treatment of RVO and AMD (37, 38). Although PDGF-BB could be induced by hypoxia and a previous study showed that combined suppression of VEGF and PDGF-BB provided superior outcomes versus suppression of VEGF alone in an early-phase clinical trial in patients with exudative AMD (39), our study showed higher levels of PDGF-BB in the AMD and RVO groups, but there was no significance.

On the other hand, many studies have reported the relationship between inflammatory stress and AMD or RVO. IL-6 is released from the lymphocytes and macrophages, as well as from the skeletal muscle cells, and acts both as a pro-inflammatory cytokine and as an anti-inflammatory myokine, depending on the stimuli (40). IL-6 might be one of the key molecules in the RPE response to oxidative stress (41). A recently study reported that IL-6 was higher in AMD patients than in cataract patients, who all also had AIDS (42). One study reported IL-6 values remaining within the normal range before and after the treatment of anti-VEGF (43). IL-8 is a main chemoattractant for neutrophils. It has been shown that intraocular IL-8 levels are higher in AMD, retinitis pigmentosa (RP), and glaucoma patients (25, 44). Also, one study showed that IL-8 was higher in AMD, and enhanced by A2E (45, 46). IL-10 is an anti-inflammatory cytokine that reduces activation of T cells. A previous study has also shown that IL-10 promotes ocular neovascularization (NV) through macrophage response to retina ischemia (47). No significant difference in IL-10 level between RP, AMD, glaucoma, and cataract patients has been found by one previous study (48). However, another study reported higher levels of IL-10 could be associated with AMD (49). Our study showed that the levels of IL-6, IL-8, and IL-10 in the aqueous of AMD groups were higher than those of controls, but only IL-10 had a significant difference. On the contrary, the levels of IL-6, IL-8, and IL-10 in the aqueous of the RVO group were significantly higher, which is consistent with previous studies (50, 51). TNF-α also plays a very important role in the pathogenesis of AMD as lower serum TNF-α levels were associated with an increase in visual acuity after anti-VEGF therapy in AMD patients (52). Bevacizumab, an anti-VEGF drug, was found to reduce the expression of IL-8 and TNF-α in RPE cells (53). Also, one study reported that TNF-α had a more than sixfold increase in RVO eyes versus controls (51). Our study showed higher levels of TNF-α in the AMD and RVO groups, significantly consistent with those of previous studies. It is interesting that the level of TNF-β in the RVO group was quite high in our study, but there was no significance. The role of TNF-β in the pathogenesis of AMD or RVO has not been reported. We might need more samples to explore the role of TNF-β in those retinal diseases.

ICAM-1, VCAM-1, IP-10, and MCP-1 are chemokines. VCAM-1 and ICAM-1 might be associated with dry-AMD (54). The inhibition of MCP-1 or ICAM-1 could protect RPE from inflammatory stress (55). A previous study suggested that IP-10 played a critical role as an antiangiogenic factor and, at the same time, as an inflammatory factor in the pathogenesis and pathophysiology of NV AMD eyes at onset and after IVA initiation (56). ICAM-1, VCAM-1, and IP-10 levels were higher in RVO patients (57, 58). The MCP-1 level in the aqueous humor of patients with RVO-ME was significantly higher compared with control and was positively correlated with the central retinal thickness (50). Diabetic ME patients with normal VEGF levels but with high levels of IL-8, IP-10, and MCP-1 showed more resistance to the anti-VEGF treatment (59). Our study showed significantly higher levels of ICAM-1 (no significance), VCAM-1, IP-10, and MCP-1 in AMD and RVO eyes, which are consistent with the results of previous studies. It also indicated the very important roles of inflammatory stress in the pathogenesis and treatment of retinal diseases, such as AMD and RVO.

## Limitations

Our samples are from the elderly, which may create a selection bias. Another limitation is the lack of serum level of irisin from the subjects. Moreover, the average age of RVO patients was relatively younger than that of the AMD and control groups. Further experiments are needed to clarify the detailed mechanisms underlying the relationship between irisin and cytokines with AMD and RVO.

## Conclusions

The metabolic factor, irisin levels in the aqueous humor are decreased in AMD and RVO eyes and show a positive correlation between irisin and MT in RVO eyes, prompting researchers to explore the relationship between irisin and macular edema. We also identified the higher expression of vascular growth factors (VEGF-A, VEGF-R1, and PDGF-BB), inflammatory cytokines (IL-6, IL-8, IL-10, and TNF-α), and chemokines (ICAM-1, VCAM-1, IP-10, and MCP-1) in AMD and RVO eyes.

## Data Availability Statement

The original contributions presented in the study are included in the article/supplementary material. Further inquiries can be directed to the corresponding author.

## Ethics Statement

The studies involving human participants were reviewed and approved by the Human Research Ethics Committee of Peking University People’s Hospital and Peking University International Hospital. The patients/participants provided their written informed consent to participate in this study.

## Author Contributions

XL and XC contributed to collection of samples, data analysis, and writing. MZ revised this manuscript. YB designed and directed the manuscript. All authors contributed to manuscript revision, read, and approved the submitted version.

## Conflict of Interest

The authors declare that the research was conducted in the absence of any commercial or financial relationships that could be construed as a potential conflict of interest.

## Publisher’s Note

All claims expressed in this article are solely those of the authors and do not necessarily represent those of their affiliated organizations, or those of the publisher, the editors and the reviewers. Any product that may be evaluated in this article, or claim that may be made by its manufacturer, is not guaranteed or endorsed by the publisher.
